# Vertical asymmetry analysis of the macular microvasculature in epiretinal membrane patients with open-angle glaucoma

**DOI:** 10.1038/s41598-023-44053-2

**Published:** 2023-10-10

**Authors:** Kee-Sup Park, Il Jung, Hyung-Bin Lim, Kook-Hyung Lee, Jung-Tae Kim, Yong-Yeon Song, Min-Woo Lee

**Affiliations:** 1 Department of Ophthalmology, Konyang University College of Medicine, Konyang University Hospital, #1643 Gwanjeo-dong, Seo-gu, Daejeon, Republic of Korea; 2 1.0 Eye Clinic, Daejeon, Republic of Korea

**Keywords:** Diseases, Medical research

## Abstract

To identify the usefulness of vertical asymmetry analysis of the retinal microvasculature in epiretinal membrane (ERM) patients accompanied by open-angle glaucoma (OAG). Subjects were divided into three groups: normal controls (group 1), patients with ERM (group 2), and patients with both ERM and OAG (group 3). Retinal nerve fiber layer (pRNFL) and ganglion cell-inner plexiform layer (GC-IPL) thicknesses, vessel density (VD), and the absolute vertical difference of pRNFL (vdRNFL), GC-IPL (vdGC-IPL), and VD (vdVD) were compared among groups. Logistic regression analysis was performed to determine the factors associated with OAG. Diagnostic accuracy based on the area under the curve (AUC) was conducted. The VD of the full area was 20.9 ± 1.2, 20.0 ± 1.9, and 18.8 ± 2.2 mm^−1^ (P < 0.001) for groups 1, 2, and 3, respectively. The vdVD differed significantly between group 2 and group 3 (P < 0.001), whereas vdRNFL (P = 0.531) and vdGC-IPL (P = 0.818) did not show a significant difference. Multivariate logistic analyses showed that average pRNFL thickness (OR 0.924, P = 0.001) and vdVD (OR 5.673, P < 0.001) were significant factors associated with OAG in ERM patients. The AUC of the vdVD was 0.81 (95% CI 0.72–0.89), and the combination of average pRNFL thickness and vdVD had the highest AUC (0.87; 95% CI 0.78–0.95; P < 0.001). ERM patients with OAG had a significantly thinner pRNFL thickness, lower macular VD, and higher vdVD than those without OAG. Average pRNFL thickness and vdVD were significant factors associated with OAG in patients with ERM. Additionally, the combination of average pRNFL thickness and vdVD showed good diagnostic performance for OAG in patients with ERM.

## Introduction

Glaucoma is progressive optic neuropathy, which is one of the leading causes of irreversible vision loss^1,2^. Because of retinal ganglion cell death and optic nerve fiber loss in glaucoma, the inner retinal layer becomes damaged as the disease progresses, as confirmed in optical coherence tomography (OCT) images showing a reduction in the inner retinal layer thickness^3^. Therefore, measurement of the inner retinal layer thickness using OCT is crucial for the diagnosis and evaluation of glaucoma. However, if accompanied by an epiretinal membrane (ERM), it may affect the thickness of the inner retinal layer and its fine measurement. Tangential traction, caused by fibrocellular proliferation on the inner retinal surface of the posterior pole area, can make the inner retina thicken; this may also cause inaccurate measurement of individual inner retinal layer thicknesses due to the ambiguous inner retina contour^4–6^. As there was a report that the prevalence of glaucoma was higher in ERM patients, early and accurate diagnosis might be important^7^. However, there are limitations in evaluating glaucoma using OCT in ERM patients.

With the development of OCT angiography (OCTA), which enables observation of the microvasculature of multiple retinal layers in a non-invasive way, impairment of the retinal microvasculature associated with glaucoma has been reported. Hong et al.^8^ demonstrated that the absolute difference between superior and inferior hemiretinal vessel area density was higher in early glaucoma, which was helpful in the diagnosis of early primary open-angle glaucoma (POAG). Chang et al.^9^ reported that the discriminant capability of macular perfusion density asymmetry for discriminating between glaucoma patients with and without visual field defects was significantly higher than that of structural asymmetry. As such, vertical asymmetry analysis of the retinal microvasculature using OCTA can be helpful for the diagnosis and evaluation of glaucoma. It may be more useful, especially when confounding factors affecting the inner retinal layer thickness, such as ERM, are involved.

The purpose of this study was to identify the usefulness of vertical asymmetry analysis of the retinal microvasculature in ERM patients accompanied by open-angle glaucoma (OAG).

## Methods

### Patients

This retrospective cross-sectional study adhered to the tenets of the Declaration of Helsinki and was approved by the Institutional Review Board/Ethics Committee of Konyang University Hospital, Daejeon, Republic of Korea (No. 2022-10-024). We reviewed the charts of patients with idiopathic ERM who visited the retina clinic of Konyang University Hospital from March 2019 to August 2022. The requirement to obtain informed consent was waived due to the retrospective nature of the study, which was approved by the Institutional Review Board/Ethics Committee of Konyang University Hospital. We recorded medical histories, best-corrected visual acuity (BCVA), intraocular pressure, spherical equivalent, and axial length. Subjects were divided into three groups: normal controls (group 1), patients with ERM (group 2), and patients with both ERM and OAG (group 3). An OAG diagnosis was confirmed by glaucoma specialists based on evidence of neural rim loss on dilated stereoscopic examination of the optic nerve, glaucomatous visual field defects apparent on standard automated perimetry (a cluster of ≥ 3 points with P < 0.05 on a pattern deviation map in at least one hemifield, including ≥ 1 point with P < 0.01; a pattern standard deviation of P < 0.05; or a glaucoma hemifield test result outside the normal limits), and/or peripapillary retinal nerve fiber layer (pRNFL) loss evident on the peripapillary assessment of spectral-domain (SD-OCT), and open angles on indentation gonioscopy, as a previous study^10^. The ERM stages were classified according to the ectopic inner foveal layer as in previous studies (stage 1, mild ERM with few anatomical modifications; stage 2, ERM with the loss of foveal depression, but well-defined all retinal layers; stage 3, continuous ectopic inner foveal layers cover the whole foveal floor with well-defined all retinal layers; stage 4, advanced ERM with complete foveal disorganization)^4,11^. A manual adjustment was performed for mild correctable segmentation errors, and all images were checked and verified by two independent observers (K.S.P. and M.W.L.). The exclusion criteria were a history of ophthalmic diseases other than idiopathic ERM and OAG, such as inflammatory eye diseases, a full-thickness macular hole, vitreomacular traction, secondary ERM, retinal vessel occlusion, secondary glaucoma, angle-closure glaucoma, a severe cataract potentially affecting the BCVA, and previous intraocular surgery excluding cataract extraction. We also excluded eyes with an axial length over 26.0 mm, eyes with ERM stage 4, in which it was impossible to segment the retina due to loss of the inner retina contour, or eyes with a BCVA lower than 20/200. If both eyes met the inclusion criteria, the eye with the higher image quality was selected for analysis.

### OCT and OCTA measurements

The ganglion cell-inner plexiform layer (GC-IPL) thickness was measured using SD-OCT (Cirrus HD OCT 6000, version 10.0; Carl Zeiss Meditec, Dublin, CA, USA) and a 512 × 128 macular cube scanning protocol, which was based on an algorithm of the Ganglion Cell Analysis module. Average, minimum, and six-sector GC-IPL thicknesses (superior, superotemporal, inferotemporal, inferior, inferonasal, and superonasal) were measured. The pRNFL thickness was measured by SD-OCT (Spectralis HRA + OCT, version 6.9.5.0; Heidelberg Engineering, Heidelberg, Germany), which acquires a 3.45 mm-diameter circle scan. The global average was calculated as the average thickness of all 768 points distributed equidistantly around the optic nerve head. Sectoral RNFL thicknesses were calculated using the averages of the points in each sector (superotemporal, temporal, inferotemporal, inferonasal, nasal, and superonasal).

OCTA images were obtained using a Cirrus 6000 HD-OCT with AngioPlex software (Carl Zeiss Meditec). A fovea-centered scan with a 3 × 3 mm^2^ area was obtained, and en-face OCTA images were automatically generated using the AngioPlex software optical microangiography algorithm. The 3 × 3 mm^2^ scan was composed of a 1 mm center and four quadrant sectors that were identical to the inner circles of the Early Treatment of Diabetic Retinopathy Study (ETDRS). The central area is a central circle with a diameter of 1 mm, the inner area is the sum of the four quadrant sectors, and the full area is a 3 mm inner circle of the ETDRS. The vessel density (VD; total length of perfused vasculature per unit area) and perfusion density (PD; total area of perfused vasculature per unit area) of the superficial vascular plexus (spanning from the internal limiting membrane to the IPL) were automatically measured by the software. The fovea avascular zone (FAZ) area was automatically measured by the software. Images with fixation loss, definite segmentation errors, motion artifacts, or a signal strength < 9 were excluded.

### Vertical asymmetry analyses of OCT and OCTA parameters

The vertical difference in GC-IPL (vdGC-IPL) was defined as the absolute difference between the superior and inferior sectors of GC-IPL thickness. The vertical difference in pRNFL (vdRNFL) was defined as the absolute difference between the superotemporal and inferotemporal sectors of pRNFL thickness. The vertical difference in VD and PD (vdVD and vdPD) was defined as the absolute difference between the superior and inferior sectors of VD and PD.

### Statistical analyses

Demographics and OCT and OCTA parameters were compared using a one-way analysis of variance followed by the post hoc Bonferroni test. The chi-square test was used to compare categorical data. Univariate and multivariate logistic regression analyses were performed to determine the factors associated with OAG in patients with ERM. Diagnostic performance for the vertical difference in OCT and OCTA parameters was conducted with an analysis of the area under the curves (AUCs). DeLong’s test was used to determine the statistical significance of differences in AUCs from various models. All statistical analyses were performed using SPSS software (version 22.0; IBM Corp., Armonk, NY, USA) and R Studio (version 1.1.453; R Foundation for Statistical Computing, Vienna, Austria).

## Results

### Demographics

Twenty-five cases were excluded because of low image quality and obvious segmentation errors. As a result, a total of 187 eyes were enrolled: 59 in group 1, 83 in group 2, and 45 in group 3. Manual adjustment was performed for 6 cases in group 2 and 3 cases in group 3. The mean age was 67.9 ± 4.1, 66.9 ± 8.4, and 69.2 ± 6.9 years in groups 1, 2, and 3, respectively (P = 0.320), and the ERM stage of 1, 2, and 3 was 41, 13, and 29 in group 2 and 14, 13, and 18 in group 3 (P = 0.169) (Table 1). Other characteristics including sex, laterality, BCVA, spherical equivalent, intraocular pressure, and axial length did not differ significantly between the three groups.

**Table 1:** Demographics and characteristics of each group.

	Group 1 (n = 59)	Group 2 (n = 83)	Group 3 (n = 45)	P value
Age (mean ± SD, years)	67.9 ± 4.1	66.9 ± 8.4	69.2 ± 6.9	0.320
Sex (man, %)	37 (62.7)	53 (63.9)	26 (57.7)	0.780
Hypertension (%)	16 (27.1)	30 (36.1)	15 (33.3)	0.695
Diabetes (%)	12 (20.3)	9 (10.8)	7 (15.6)	0.109
Laterality (right, %)	26 (44.1)	44 (53.0)	20 (44.4)	0.721
BCVA (mean ± SD, logMAR)	− 0.02 ± 0.06	0.16 ± 0.18	0.13 ± 0.15	**< 0.001**
SE (mean ± SD, diopter)	+ 0.27 ± 1.12	+ 0.06 ± 1.58	− 0.16 ± 1.63	0.194
IOP (mean ± SD, mmHg)	14.0 ± 2.5	13.3 ± 2.7	13.4 ± 3.1	0.092
Axial length (mean ± SD, mm)	23.8 ± 0.8	23.7 ± 0.9	24.0 ± 0.6	0.132
MD (mean ± SD, dB)	n/a	n/a	− 7.03 ± 5.72	n/a
PSD (mean ± SD, dB)	n/a	n/a	6.23 ± 3.73	n/a
VFI (mean ± SD, %)	n/a	n/a	83.2 ± 16.5	n/a
ERM stage (1:2:3)	n/a	41: 13: 29	14 : 13: 18	0.169
CMT (mean ± SD, μm)	249.9 ± 18.7	366.8 ± 69.6	364.9 ± 61.8	** < 0.001**

Values in boldface (P < 0.050) are statistically significant.

*SD* standard deviation, *BCVA* best-corrected visual acuity, spherical equivalent, *IOP* intraocular pressure; mean deviation, *PSD* pattern standard deviation, *VFI* visual field index, *ERM* epiretinal membrane, *CMT* central macular thickness.

### OCT and OCTA paramerters

The average GC-IPL thickness was 84.9 ± 10.6, 66.9 ± 22.3, and 65.5 ± 18.9 μm in groups 1, 2, and 3, respectively (P < 0.001) (Table 2). In post hoc analysis, the average GC-IPL thickness of group 1 was significantly thicker than that of group 2 and group 3 (both P < 0.001), and that of group 2 was not significantly different from that of group 3 (P = 0.484). The average pRNFL thickness was 96.6 ± 8.2, 101.1 ± 11.0, and 85.3 ± 21.5 μm in groups 1, 2, and 3, respectively (P < 0.001), and the difference was significant. In post hoc analysis, the pRNFL thickness of group 3 was significantly thinner than that of group 1 and group 2 (both P < 0.001). Sectoral pRNFL thicknesses were also significantly different among the groups, except for the nasal sector.

**Table 2:** Ganglion cell-inner plexiform layer and peripapillary retinal nerve fiber layer thicknesses in each group.

	Group 1	Group 2	Group 3	P value
GC-IPL				
Average	84.9 ± 10.6	69.9 ± 22.3	65.5 ± 18.9	** < 0.001**
Minimum	80.0 ± 6.9	45.1 ± 26.2	40.1 ± 23.2	** < 0.001**
Sector				
Superior	83.7 ± 6.2	69.1 ± 33.5	66.0 ± 26.8	**0.003**
Superotemporal	82.1 ± 5.0	75.4 ± 27.9	65.8 ± 24.3	**0.006**
Inferotemporal	83.3 ± 5.1	74.3 ± 28.8	64.1 ± 24.0	**0.001**
Inferior	81.2 ± 5.5	61.0 ± 27.4	58.3 ± 25.7	** < 0.001**
Inferonasal	83.1 ± 5.5	67.4 ± 22.3	69.1 ± 23.6	** < 0.001**
Superonasal	85.2 ± 5.8	71.9 ± 25.8	71.2 ± 17.6	**0.001**
pRNFL				
Average	96.6 ± 8.2	101.1 ± 11.0	85.3 ± 21.5	** < 0.001**
Sector				
Superotemporal	120.3 ± 15.8	127.8 ± 19.0	97.8 ± 28.6	** < 0.001**
Temporal	70.3 ± 10.1	91.3 ± 17.5	78.8 ± 17.0	** < 0.001**
Inferotemporal	121.8 ± 16.3	146.0 ± 23.3	100.6 ± 38.1	** < 0.001**
Inferonasal	91.8 ± 38.4	104.4 ± 23.4	91.8 ± 38.4	**0.004**
Nasal	70.6 ± 10.6	70.3 ± 11.8	73.0 ± 35.6	0.347
Superpnasal	104.8 ± 20.9	105.9 ± 21.2	92.5 ± 28.5	**0.002**

Values in boldface (P < 0.050) are statistically significant.

All values are expressed as the mean ± standard deviation (μm).

*GC-IPL* ganglion cell-inner plexiform layer, *Prnfl* peripapillary retinal nerve fiber layer.

**Figure 1:**
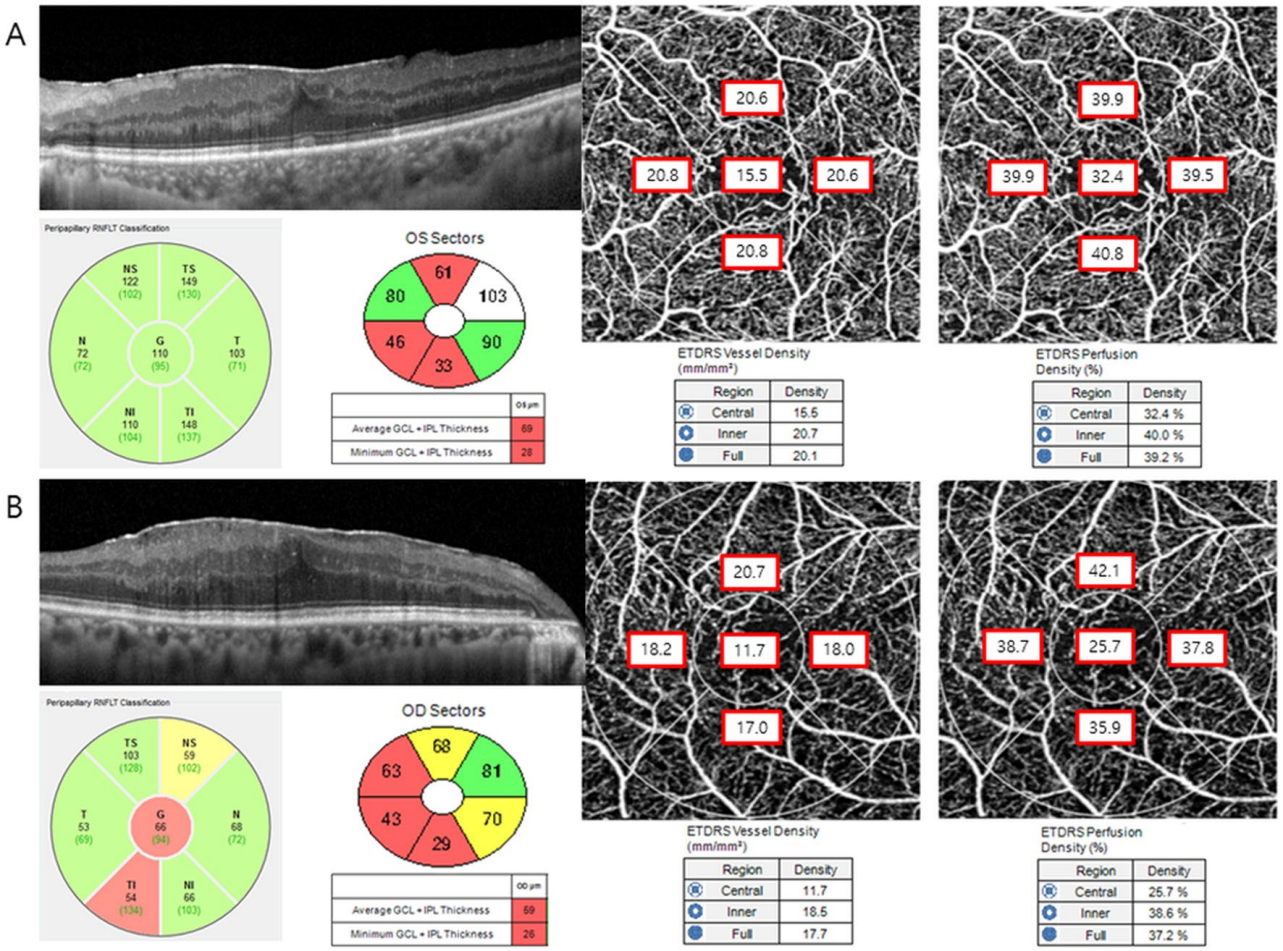
Representative cases of B-scan images, peripapillary retinal nerve fiber layer thickness (pRNFL), ganglion cell-inner plexiform layer (GC-IPL) thickness, vessel density (VD), and perfusion density (PD) in group 1 (**A**) and group 2 (**B**).

The VD of the full area was 20.4 ± 1.2, 20.0 ± 1.9, and 18.8 ± 2.2 mm^−1^ in groups 1, 2, and 3, respectively (P < 0.001), and the difference was significant (Table 3). In post hoc analysis, the VD of group 3 was significantly lower than that of group 1 (P < 0.001) and group 2 (P = 0.004). The VDs of the inner area and superior, temporal, and inferior sectors were also significantly different between the groups. The PD of the full area was 37.5 ± 1.8, 38.9 ± 3.2, and 37.2 ± 5.1% for groups 1, 2, and 3, respectively (P = 0.019). The PDs of the inner area, superior, and inferior sectors were also significantly different between the groups (Fig. 1). The FAZ area was significantly different among the groups (P < 0.001), but it did not remain significant between group 2 and group 3 in the post hoc analysis (P = 0.987).

**Table 3:** Optical coherence tomography angiography parameters in each group.

	Group 1	Group 2	Group 3	P value
Vessel density (mm^−1^)				
Full area	20.4 ± 1.2	20.0 ± 1.9	18.8 ± 2.2	** < 0.001**
Inner area	22.3 ± 1.2	20.7 ± 1.9	19.4 ± 2.2	** < 0.001**
Central area	9.8 ± 2.6	15.8 ± 17.3	14.0 ± 4.7	**0.032**
Sector				
Superior	22.4 ± 1.4	20.8 ± 2.0	19.4 ± 2.8	** < 0.001**
Temporal	22.0 ± 1.3	20.3 ± 2.7	18.9 ± 2.3	** < 0.001**
Inferior	22.3 ± 1.4	20.7 ± 1.9	19.1 ± 2.6	** < 0.001**
Nasal	22.5 ± 1.5	20.9 ± 2.1	20.2 ± 3.0	** < 0.001**
Perfusion density (%)				
Full area	37.5 ± 1.8	38.9 ± 3.2	37.2 ± 5.1	**0.019**
Inner area	40.1 ± 1.7	40.2 ± 2.7	38.4 ± 4.7	**0.008**
Central area	17.2 ± 4.5	26.2 ± 7.5	26.8 ± 9.6	** < 0.001**
Sector				
Superior	40.3 ± 2.5	40.5 ± 3.3	38.4 ± 5.8	**0.023**
Temporal	39.9 ± 2.2	39.2 ± 5.0	37.9 ± 7.2	0.185
Inferior	40.0 ± 2.4	40.7 ± 2.9	38.1 ± 5.8	**0.002**
Nasal	40.2 ± 2.6	40.6 ± 3.7	39.7 ± 6.0	0.564
FAZ area	0.32 ± 0.09	0.17 ± 1.12	0.17 ± 0.13	** < 0.001**

Values in boldface (P < 0.050) are statistically significant.

All values are expressed as the mean ± standard deviation.

*FAZ* fovea avascular zone.

### Vertical asymmetry analysis of OCT and OCTA parameters

The vdGC-IPL was 3.6 ± 2.3, 19.8 ± 23.3, and 17.6 ± 17.6 μm in groups 1, 2, and 3, respectively (P < 0.001), and the vdRNFL was 11.5 ± 8.7, 24.3 ± 18.4, and 27.9 ± 19.6 μm (P < 0.001) (Table 4). In post hoc analysis, both vdGC-IPL (P = 0.818) and vdRNFL (P = 0.531) were not significantly different between group 2 and group 3. The vdVD of groups 1, 2, and 3 was 1.10 ± 0.89, 1.05 ± 0.75 and 2.81 ± 1.99 mm^−1^ (P < 0.001) and the vdPD was 2.13 ± 2.25, 2.55 ± 2.30, and 4.73 ± 3.79% (P < 0.001), respectively. In post hoc analysis, both vdVD and vdPD of group 3 were significantly higher than those of group 1 and group 2 (all P < 0.001).

**Table 4:** Vertical difference in optical coherence tomography and optical coherence tomography angiography parameters in each group.

	Group 1	Group 2	Group 3	P value
vdGC-IPL (μm)	3.6 ± 2.3	19.8 ± 23.3	17.6 ± 17.6	** < 0.001**
vdRNFL (μm)	11.5 ± 8.7	24.3 ± 18.4	27.9 ± 19.6	** < 0.001**
vdVD (mm^−1^)	1.10 ± 0.89	1.05 ± 0.75	2.81 ± 1.99	** < 0.001**
vdPD (%)	2.13 ± 2.25	2.55 ± 2.30	4.73 ± 3.79	** < 0.001**

Values in boldface (P < 0.050) are statistically significant.

All values are expressed as the mean ± standard deviation.

*vdGC-IPL* absolute vertical difference in ganglion cell-inner plexiform layer thickness, *vdRNFL* absolute vertical difference in retinal nerve fiber layer thickness, *vdVD* absolute vertical difference in vessel density, *vdPD* absolute vertical difference in perfusion density.

### Logistic regression analyses and diagnostic accuracy based on the AUC

In univariate logistic analyses, pRNFL (OR 0.918, P < 0.001), VD of the full area (OR 0.764, P = 0.014), vdVD (OR 3.244, P < 0.001), and vdPD (OR 1.294, P < 0.001) were significantly associated with group 2 (Table 5). Multivariate analyses showed that pRNFL (OR 0.924, P = 0.001) and vdVD (OR 5.673, P < 0.001) were significant factors.

**Table 5:** Univariate and multivariate logistic regression analyses to determine the factors associated with glaucoma in patients with epiretinal membrane.

	Univariate			Multivariate		
	B	OR (95% CI)	P value	B	OR (95% CI)	P value
Age	0.036	1.037 (0.986, 1.091)	0.162			
Sex	− 0.857	0.425 (0.190, 0.950)	**0.037**	0.140	1.150 (0.322, 4.102)	0.830
BCVA	− 1.017	0.362 (0.030, 4.404)	0.425			
SE	− 0.085	0.919 (0.721, 1.171)	0.493			
IOP	0.022	1.022 (0.890, 0.174)	0.760			
Axial length	0.513	1.670 (1.023, 2.727)	**0.040**	0.384	1.469 (0.735, 2.937)	0.277
ERM stage	0.321	1.379 (0.885, 2.147)	0.156			
Average GC-IPL	0.003	1.003 (0.979, 1.028)	0.809			
vdGC-IPL	− 0.001	0.999 (0.979, 1.019)	0.908			
Average pRNFL	− 0.085	0.918 (0.884, 0.954)	** < 0.001**	− 0.079	0.924 (0.880, 0.969)	**0.001**
vdRNFL	0.018	1.018 (0.995, 1.041)	0.127			
Full VD	− 0.269	0.764 (0.616, 0.947)	**0.014**	− 0.253	0.776 (0.561, 1.075)	0.128
vdVD	1.177	3.244 (1.969, 5.344)	** < 0.001**	1.736	5.673 (2.233, 14.412)	** < 0.001**
Full PD	− 0.113	0.893 (0.799, 0.998)	**0.046**	0.118	1.125 (0.945, 1.341)	0.185
vdPD	0.261	1.298 (1.126, 1.498)	** < 0.001**	− 0.307	0.735 (0.530, 1.021)	0.067
FAZ area	− 0.249	0.779 (0.027, 22.320)	0.884			

Values in boldface (P < 0.050) are statistically significant.

*BCVA* best-corrected visual acuity, *SE* spherical equivalent, *IOP* intraocular pressure, *GC-IPL* ganglion cell-inner plexiform layer, *vdGC-IPL* absolute vertical difference in GC-IPL thickness, *pRNFL* peripapillary nerve fiber layer, *vdRNFL* absolute vertical difference in RNFL thickness, *VD* vessel density, *vdVD* absolute vertical difference in VD, *PD* perfusion density, *vdPD* absolute vertical difference in PD, *FAZ* fovea avascular zone.

**Figure 2:**
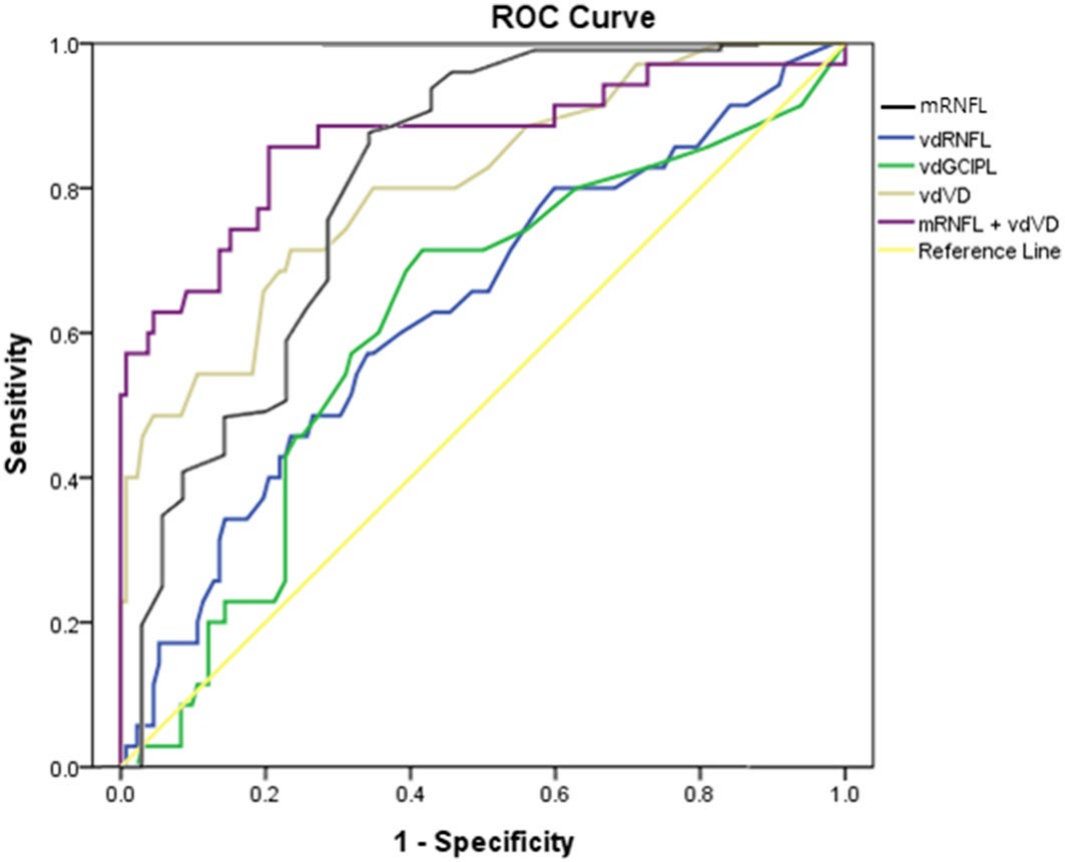
Receiver operating characteristic curves for the mean peripapillary retinal nerve fiber layer (mRNFL) thickness, the absolute vertical difference in vessel density (vdVD), RNFL (vdRNFL), ganglion cell-inner plexiform layer thickness (vdGC-IPL), and the combination of mRNFL and vdVD. The area under the curve (95% CI) was 0.80 for mRNFL (0.71–0.90), 0.81 for vdVD (0.72–0.89), 0.63 for vdRNFL (0.53–0.74), 0.62 for vdGC-IPL (0.51–0.72), and 0.87 for combination of mRNFL and vdVD (0.78–0.95). The dashed diagonal line shows the line of no discrimination.

The diagnostic accuracy as measured with the AUC for average pRNFL was 0.80 (95% CI 0.71–0.90; P < 0.001); for average GC-IPL 0.63 (95% CI 0.53–0.73; P = 0.016); for VD of the full area 0.75 (95% CI 0.66–0.84; P < 0.001); for vdVD 0.81 (95% CI 0.72–0.89; P < 0.001); for vdPD 0.67 (95% CI 0.56–0.79; P = 0.003); for vdRNFL 0.63 (95% CI 0.53–0.74; P = 0.016); and for vdGC-IPL 0.62 (95% CI 0.51–0.72; P = 0.037) (Fig. 2). Of the parameters, the combination of average pRNFL thickness and vdVD had the highest AUC (0.87; 95% CI 0.78–0.95; P < 0.001), which was significantly greater than all other parameters (all P < 0.001).

## Discussion

In this study, we evaluated the inner retinal layer thickness including macular GC-IPL and pRNFL, macular VD and PD, and the vertical asymmetry of those parameters in normal controls, patients with only ERM, and those with both ERM and OAG. We found that ERM patients with OAG had a significantly thinner pRNFL thickness, lower macular VD and PD, and higher vdVD and vdPD than those without OAG, whereas GC-IPL thickness, vdGC-IPL, and vdRNFL did not show significant differences. Average pRNFL thickness and vdVD were significant factors associated with OAG in patients with ERM. Additionally, the combination of average pRNFL thickness and vdVD showed good diagnostic performance for OAG in patients with ERM.

Whereas macular GC-IPL thickness was not significantly different between group 2 and group 3, pRNFL thickness showed a significant difference and a relatively good diagnostic power in this study. The ERM is often thicker around the macula than around the peripapillary area, which results in less of an impact of ERM on the pRNFL thickness than on the macular GC-IPL thickness. Although pRNFL thickness may be less susceptible to ERM than macular GC-IPL thickness, it can be inappropriate to evaluate glaucoma relying solely on the pRNFL thickness in ERM patients. Previous studies reported that ERM can increase the pRNFL thickness by contractile force on the internal limiting membrane and RNFL edema due to retinal vascular deformation, which can obscure diffuse or localized thinning of the pRNFL^5,12,13^. Vertical asymmetry of the GC-IPL and pRNFL thicknesses, which have been reported to have good glaucoma diagnostic ability, did not also show a significant difference between group 2 and group 3 and was not a significant factor associated with OAG in ERM patients in this study^14–16^. Therefore, depending solely on the analysis of inner retinal layer thickness using OCT may delay the diagnosis of OAG or result in missing OAG progression in ERM patients.

Previous studies reported a reduction in retinal microvasculature using OCTA in glaucoma^17–19^. Yarmohammadi et al.^18^ showed that the age-adjusted mean VD was significantly lower in OAG eyes compared with glaucoma suspects and healthy eyes. Scripsema et al.^17^ reported that the perfused capillary density in POAG (32.24 ± 6.76%) and normal tension glaucoma (37.75 ± 3.52%) patients was significantly lower compared to that in normal subjects (42.99 ± 1.81%). Our study also showed that group 3 had a significantly lower macular VD and PD than group 1 and group 2. Although the ERM can affect the retinal microvasculature by deformation of the foveal avascular zone and increased vascular tortuosity, microvascular impairment caused by OAG may be reflected in the OCTA image, even in the presence of ERM. Therefore, analyses of the macular microvasculature using OCTA can be an effective tool for OAG evaluation in ERM patients.

Smith et al.^20^ reported that values for PD asymmetry yielded a significant correlation with mean deviation and GCL thickness, and improved the separation between patients with glaucoma and healthy controls. Another study indicated that age-adjusted mean predicted asymmetry values of peripapillary vessel area density were significantly higher in mild, moderate, and severe POAG compared to healthy controls^8^. Our study revealed that group 3 had a significantly higher vdVD and vdPD than group 1 and group 2, consistent with previous studies. Whereas ERM usually affects the general area of macular microvasculature, glaucoma affects a relatively local area, which would make the analysis of macular microvasculature asymmetry useful for the evaluation of glaucoma in ERM patients. However, although the stage of ERM, which may affect the microvasculature, was not also a significant factor associated with glaucoma in patients with ERM, physicians should be careful in the interpretation of asymmetry analysis using OCTA in the case of biased ERM located either above or below the macula, or advanced glaucoma with diffuse inner retinal thinning^21^.

A previous study reported that the AUC of macular PD asymmetry for discriminating between preperimetric glaucoma and perimetric glaucoma was 0.86, and the AUC of macular VD asymmetry for discriminating between healthy and perimetric glaucoma was 0.88^9^. Another study reported that the AUC of the perfusion asymmetry index for glaucoma diagnosis was 0.83 (95% CI 0.77–0.90)^20^. Our study showed that the AUC of the vdVD for OAG diagnosis in ERM patients was 0.81 (0.72–0.90), which is comparable with previous studies including patients without ERM. On the other hand, vdRNFL and vdGC-IPL, which are known to have good diagnostic accuracy for glaucoma, had relatively low diagnostic accuracy in ERM patients. Therefore, vdVD can be an effective clue for the diagnosis of OAG in ERM patients. Additionally, the average pRNFL thickness, which is less affected by ERM than GC-IPL thickness, was a significant factor associated with OAG in ERM patients and showed good diagnostic accuracy as well as the vdVD. Hong et al.^8^ reported that the combination of pRNFL thickness and hemiretinal difference in vessel area density showed a significantly greater AUC than pRNFL thickness alone for discriminating POAG from healthy eyes (0.859 vs 0.847, P = 0.049). Our study also showed that the combination of average pRNFL thickness and vdVD had the highest AUC for discriminating OAG among various parameters in ERM patients. Interpreting the two parameters together, average pRNFL thickness and vdVD would be effective in diagnosing OAG in ERM patients.

This study had several limitations. First, the retrospective nature of the study inevitably introduced some selection bias. Second, relatively fewer patients with advanced glaucoma were included in the study. Additional studies including a larger number of patients with a broad spectrum of disease severity are needed to analyze the diagnostic power of vdVD according to disease severity in the future. Third, although we excluded images with definite segmentation errors and patients with severe ERM showing ambiguous margins between the inner retinal layer, fine segmentation errors could not be avoided due to the irregular inner retinal contour in ERM patients. Fourth, we could not include the glaucoma patients without ERM for which more accurate results might be obtained. The strength of our study is that we presented an effective tool for evaluating OAG in patients with an important confounding factor, ERM, which has not been reported to date. Additionally, we included OCTA images with a high signal strength above 9 for accurate analysis.

In conclusion, ERM patients with OAG had a significantly thinner pRNFL than those without OAG, whereas the GC-IPL thickness was not significantly different. Although ERM would affect the peripapillary area less than the macula, both vdGC-IPL and vdRNFL were not significantly different between the groups. The macular VD and PD were significantly lower, and the vdVD and vdPD were significantly higher in ERM patients with OAG. Additionally, the vdVD showed good diagnostic accuracy for OAG in ERM patients based on AUC analysis. The impairment of macular microvasculature caused by OAG would be reflected on OCTA images even with an important confounding factor, ERM. The average pRNFL thickness and vdVD were significant factors associated with OAG, and the combination of them yielded the highest AUCs of all parameters for discriminating OAG in ERM patients. We expect that these findings will help physicians evaluate OAG in patients with ERM.

## Data Availability

The datasets used and/or analyzed during the current study are available from the corresponding author on reasonable request.
